# Day and night use of habitats by northern pintails during winter in a primary rice-growing region of Iberia

**DOI:** 10.1371/journal.pone.0220400

**Published:** 2019-07-25

**Authors:** Manuel Parejo, Jorge S. Gutiérrez, Juan G. Navedo, Andrea Soriano-Redondo, José M. Abad-Gómez, Auxiliadora Villegas, Casimiro Corbacho, Juan M. Sánchez-Guzmán, José A. Masero

**Affiliations:** 1 Conservation Biology Research Group, Department of Anatomy, Cell Biology and Zoology, Faculty of Sciences, University of Extremadura, Badajoz, Spain; 2 Centro de Estudos do Ambiente e do Mar (CESAM), Departamento de Biologia Animal, Faculdade de Ciências da Universidade de Lisboa, Lisboa, Portugal; 3 Instituto de Ciencias Marinas y Limnológicas, Universidad Austral de Chile, Valdivia, Chile; 4 Centre for Ecology and Conservation, College of Life and Environmental Sciences, University of Exeter, Cornwall, United Kingdom; University of California Los Angeles, UNITED STATES

## Abstract

Loss of natural wetlands is a global phenomenon that has severe consequences for waterbird populations and their associated ecosystem services. Although agroecosystems can reduce the impact of natural habitat loss, drivers of use of such artificial habitats by waterbirds remain poorly understood. Using the cosmopolitan northern pintail *Anas acuta* as a model species, we monitored home-range and fine-scale resource selection across the agricultural landscape. Individuals were tracked using GPS-GSM transmitters, and a suite of environmental and landscape features were measured throughout the winter seasons. Spatial patterns of habitat use were analysed using generalized linear mixed effect models by integrating field-observations with GPS telemetry. All birds used rice fields as foraging grounds at night and commuted to an adjacent reservoir to roost during daylight. Home-ranges and maximum foraging distances of nocturnally foraging birds increased with decreasing availability of flooded fields, and were positively correlated with moonlight levels. Birds selected flooded rice paddies (water depth range: 9–21 cm) with standing stubble and substrate with pebbles smaller than 0.5 cm in diameter. Density of rice seeds, rice paddy size, and other environmental and landscape features did not emerge as significant predictors. Our findings indicate that nocturnal foraging of northern pintails within rice fields is driven primarily by straw manipulation, water level and substrate pebble size. Thus, the presence of standing stubble in flooded paddies with soft bottoms should be prioritized to improve foraging areas for dabbling ducks. These management procedures in themselves would not increase economic costs or affect rice production and could be applied for dabbling-duck conservation throughout the world.

## Introduction

Globally, natural wetlands have lost around 64–71% of their area since the beginning of the 20th century [1]. In North America and Europe the rate of wetland loss has decreased or remains constant, but in many other regions, such as Asia, natural wetlands are disappearing at alarming rates [2]. Direct human alterations, often in conjunction with climate change, make natural wetlands one of the most threatened habitats on the planet [3,4]. The loss or degradation of these aquatic ecosystems has a great impact on biodiversity conservation, given the numerous plant, invertebrate and vertebrate species associated only with wetlands [5,6]. Even though the ecosystem functions of natural wetlands cannot be replaced by human-made wetlands, the latter can provide suitable habitats that partly mitigate the impact of wetland loss on aquatic biota (e.g. [7]).

Flooded rice fields occupy over 1% of the Earth's ice-free land surface [8]. The high number of rice (*Oryza sativa*) varieties has enable its growth in every continent (except Antarctica), spanning 163 million hectares from 50° N to 40° S [9]. These flooded agricultural fields are often classified as functional wetlands for many waterbird groups (e.g. waterfowls, shorebirds or cranes) [10,11], and they have a recognized potential to contribute to the conservation of wetland biota worldwide [12–14].

Most waterbird species depend on wetlands throughout their life cycle. Waterbird use of rice fields has increased as natural wetlands continue to decline (e.g. [15]), and currently many migratory waterbird species on several flyways depend on them [16]. Thus, there is a growing interest in how to manage rice fields in order to increase their value for waterbirds (e.g. [17–19]), which is especially relevant in the case of duck species given the important ecosystem services they provide [20,21].

Generally, migratory dabbling ducks forage in rice fields at night and rest in reservoirs or lakes nearby during the day (e.g. [22–24]). This nocturnal regime has hampered the study of micro- and macro-habitat use and selection in rice fields by dabbling ducks, which is imperative to design appropriate management strategies for these species (see [25–27]). To our knowledge, no study to date has assessed the factors influencing the selection of nocturnal foraging areas by dabbling ducks and other waterbirds within their home-range, i.e. at a relevant scale [28]. The only studies available are based on direct censuses at night using spotlights or light amplifiers [29,30].

Here, we evaluated fine-scale use and selection of rice field areas by nocturnally foraging dabbling ducks in one of the most important areas for rice production in Western Europe (Extremadura, SW Spain; [31]). Specifically, we focused on the northern pintail *Anas acuta* as a model—a cosmopolite and widely distributed species, present in North America, Asia, Europe and North of Africa [32]. To this end, we tagged several individuals with GPS (Global Positioning Systems) solar powered devices and used resource selection functions (RSF; [33]) at fine-scale spatiotemporal resolution. A RSF is a function of characteristics measured on resource units such its value for a unit is proportional to the probability of that resource to be used by an organism [33]. Our main goal was to identify the post-harvest treatments and environmental and landscape variables that determine the home-range and the selection of nocturnal foraging areas by dabbling ducks during the winter. We predicted no difference in proportional use of ploughed vs. unploughed rice fields by northern pintails following harvest of rice, and that most northern pintails feed at night.

## Materials and methods

### Study area

**Fig 1:**
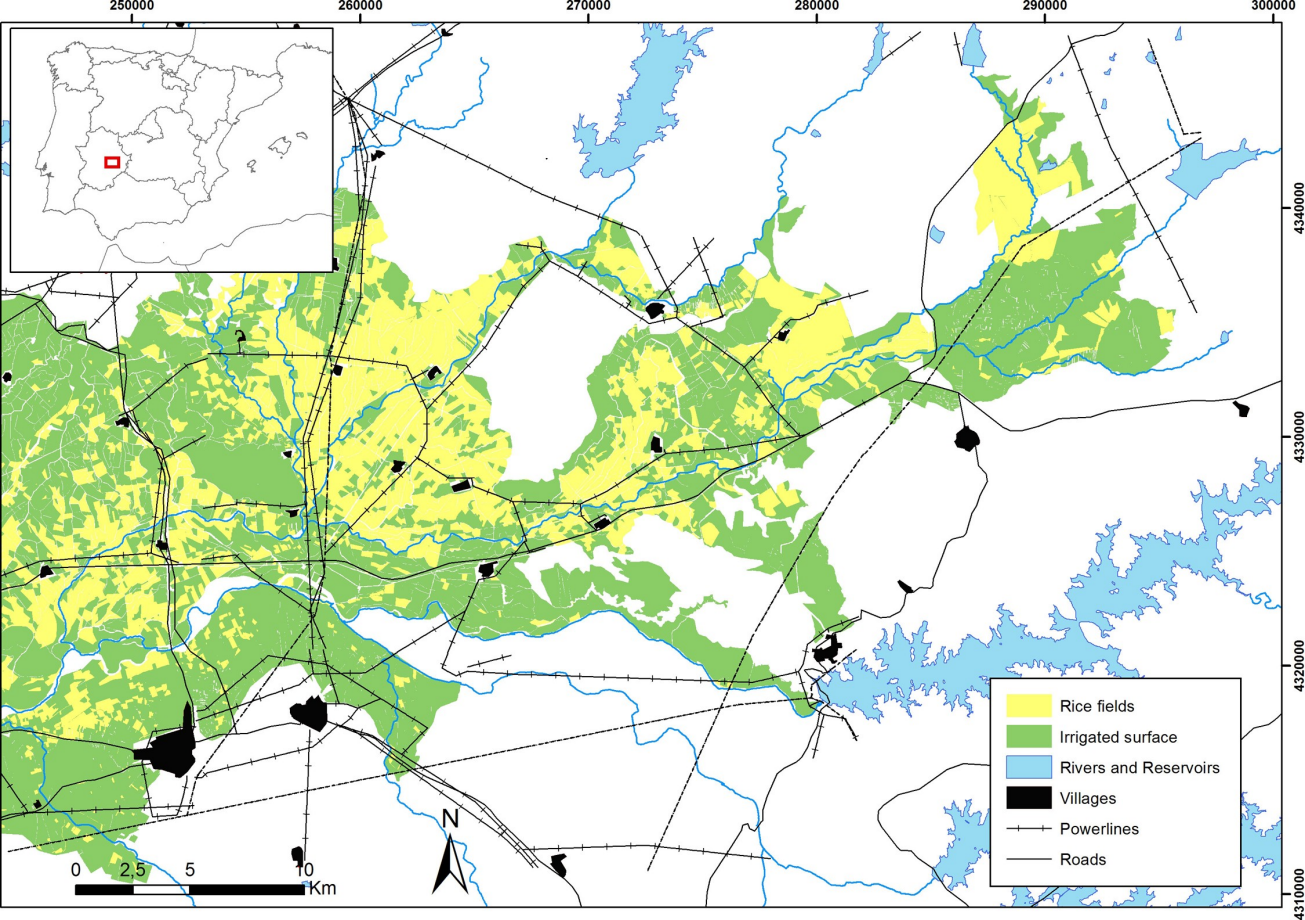
Study area (Extremadura rice fields; SW Spain) showing reservoirs, rice fields, main infrastructures and urban areas. The asterisk indicate the reservoir (Gargáligas) used by northern pintails as roosting site during daylight.

We studied rice fields and a large reservoir in southwest Spain within the Guadiana river basin (39°N, 6°W; Fig 1). This approximately 25,000 ha region is a main wintering area for dabbling ducks (e.g., northern pintail, green-winged teal *Anas crecca*, northern shoveler *Anas clypeata* [34]). This prime agricultural landscape is characterized by a marked horizontality and topographic homogeneity, and therefore, the climatic features (winters with mild temperatures) remain uniform throughout the area [34,35]. In Extremadura management of rice fields for waterbirds is not yet implemented, and while northern pintails are not hunted, other dabbling duck species such as green-winged teals and northern shovelers are permitted to be hunted during the winter.

**Fig 2:**
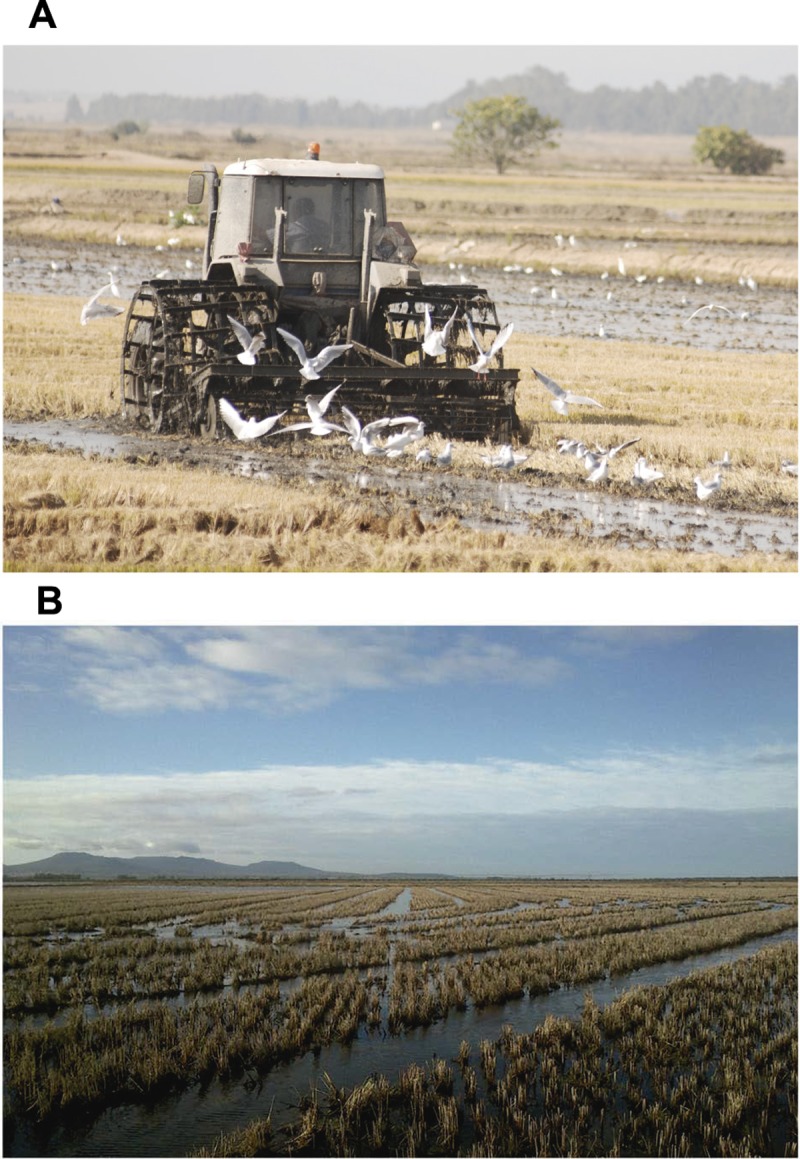
(A) Farmer ploughing a rice paddy after fall rains. Standing stubble is depicted in background, whereas in the foreground the paddy is ploughed and partly flooded with stubble incorporated into soil. (B) Flooded rice paddy with standing stubble; the stubble-free furrows created by the harvester are visible.

Extremadura rice fields are a continuum that occupies thousands of hectares (Fig 1), divided into paddies surrounded by a raised earthen levee (usually < 0.5 m high). The average size of a paddy is 2.4 ± 0.2 ha, and most of them (> 80%) range from 1 to 4 ha. Rice paddies are owned by individuals and cooperatives who apply different treatments after harvest in September–October. Similar to worldwide practices, following fall rains the partly or fully flooded fields are ploughed to incorporate the rice stubble into the soil to enhance decomposition (see Fig 2A) [36]. Many paddies, however, are not ploughed (see Fig 2B) and standing stubble (25–45 cm high) remains until paddies are prepared for planting in spring. Under these practices rice fields may remain flooded or dry depending on status of drainage channels and rainfall. Burning of stubble is prohibited.

### Tag deployment and tracking individuals

In early December, we cannon-netted twelve adult northern pintails (six individuals in 2012 and six new individuals in 2014; 9 females and 3 males in total) at Gargáligas reservoir (Fig 1). This reservoir is used as a roosting site during daylight by over 7,000 individuals (~ 1% of the population from the East Atlantic Flyway; [34]). Upon capture, all individuals were ringed, weighted and measured.

Northern pintails were tagged with GPSs using Teflon harnesses [37]. The six devices deployed in 2012 were GPS-GSM (Global System for Mobile Communications), model Duck-3 (35 g; Ecotone Telemetry, Gdynia, Poland) and the six deployed in 2014 were model GiPSy-4 (25 g; Technosmart, Roma, Italy). The mass of the devices represented 2–4% of body mass in early winter. Duck-3 devices collected locations every 2 h, and sent the data when GSM signal was available and at least five positions had been recorded. GiPSy-4 devices recorded three positions during daylight (dawn, midday, dusk) and hourly positions at night; positions were downloaded using a base station that communicated with tags through the wireless ZigBee placed within 1 km range when dabbling ducks were resting in the reservoir (this technology requires that birds to be relatively immobile) [38,39]. Both models had a location error < 20 m (average distance between consecutive locations of stationary devices). GPS devices provide a fine spatial and temporal resolution to describe bird movement patterns (e.g. [40,41]), and they allow us to understand how individuals perceive and react to environmental changes [42]. All birds tagged on 2012 with the model Duck-3 started the north migration successfully, but all they died by hunting activities out of Iberia before returning to Extremadura rice fields [37]. We also have no evidence that the birds equipped with the model GiPSy-4 returned to our study area. All tagging and field work procedures occurred under the permit CNO103/11OT from the Dirección General de Medio Ambiente de Extremadura. Tagging described in this permit, and used in this manuscript, were evaluated by a committee on the base of conservation and ethical considerations.

### Home-range

For each individual, space use was estimated weekly throughout the season (December-February). Home-range was defined as 100% of the minimum convex polygon (MCP), the best estimator to determine the exploratory activity of an individual [43]. We used R (ver. 2.12.0, R Development Core Team 2010), the Geospatial Modelling Environment (GME; [44]) and ArcGIS (ver. 10.1, Esri, Redlands, CA, U.S.A.) to calculate the weekly MCPs. The latter were calculated by considering those GPS locations recorded throughout the week (7–8 locations per night, at least one hour apart, and 3 locations during daylight—dawn, midday and dusk). Maximum foraging distance, i.e. the longest distance between the foraging positions (10 pm—2 am) and the roosting positions (12–4 pm) was calculated daily and then averaged weekly, using Euclidean distance tool in ArcGIS [45].

We examined how fluctuations in food abundance, flooded area, weather, and moon brightness could affect home-range area used by northern pintails. The proportion of flooded rice paddies was estimated following Santiago-Quesada et al. [46]. We established four transects (10–15 km) across the rice fields within the 20 km radius (~ 12,000 ha) of the main roosting site, which were visited twice a week from December to March. This 20 km radius was the maximum distance covered by radio tagged northern pintails, green-winged teals and northern shovelers when commuting between roosting and nocturnal foraging sites (unpublished data from radio tagged birds in Extremadura; Conservation Biology Research Group 2011). The percentage of flooded rice paddies with stubble, percentage of flooded and ploughed rice paddies (straw incorporated into the soil), and percentage of flooded rice paddies (independently of straw manipulation) were estimated from these transects.

Food abundance was estimated as the density of rice seeds in rice paddies within that 20 km radius. Data from stable isotope analysis showed rice seeds left on the ground after harvest are the main food source for dabbling ducks in Extremadura [31]. Every week, we randomly sampled 25 flooded rice paddies assumed to be available (see below) for foraging dabbling ducks. Using a soil core sampler (7.5 cm diameter, 10 cm depth), we sampled the sediment of each rice paddy at five randomly selected points and used the mean values in the analyses [12]. Samples were stored and preserved at—24°C for later analysis. In the laboratory, soil cores were defrosted and sieved (mesh size = 1 mm), and the number of rice seeds was counted [31,46].

Finally, daily records of the moonlight brightness (percentage of full moon) and weather (rain, wind speed, and minimum temperature) were taken from the ‘Observatorio Astronómico Nacional’ [47] and from the weather station in the Gargáligas reservoir (Confederación Hidrográfica del Guadiana), respectively. These values were averaged weekly.

### Resource selection

RSF provide an excellent framework to understand the distribution of organisms and they are essential to design and develop successful management and conservation strategies [48–50]. Each rice paddy was assumed to be an experimental unit [12]. To test whether northern pintails preferred certain rice paddies over others, we developed RSFs using a use vs. availability design (e.g. [51]). We defined available rice paddies as those that contained surface water at the time of selection. Every week, we selected four random locations within the MCP of a given individual and established a relationship between the available and the used rice paddies of 3:1 [52,53]. To avoid telemetry errors and confounding effects on the estimation of the probability of occurrence, these random locations were > 20 m away from used rice paddies [54].

Used rice paddies were selected considering those GPS locations recorded between 10 pm and 2 am, since the maximum foraging activity of dabbling ducks occurs during this time ([30,55]; personal observations). In general (> 80% of cases), after 8 pm tagged northern pintails remained in the same rice paddy for several consecutive hours (see *Results*).

For each paddy (both used and not used), we recorded food abundance (see above), pebble size, water depth, paddy size, straw manipulation, distance to diurnal roosting site (Gargáligas), and distance to the closest power line, paved road and urban area. The pebbles found in the soil corers used for estimating food abundance were classified into three different categories according to their diameter (< 0.5 cm, 0.5–1.0 cm, and > 1.0 cm; [56]), because pebble size can affect foraging in dabbling ducks [57,58]. We also measured water depth at five different points following the diagonal of the rice paddy and maintaining a constant distance between sampling points [30]. Dabbling ducks can distribute through flooded rice fields depending on water depth [59,60]. To estimate the size of the rice paddies and landscape variables, we used raster images of the study area [35]. The distance to the closest urban area, paved road and power line were used as measures of human disturbances and were estimated from the centre of each rice paddy [46,61,62]. All distance variables were calculated using Euclidean Distance tool in ArcGIS [63,64]

### Statistical analysis

We used generalized linear mixed models (GLMM) to test the effect of several variables on weekly MCP (ha) and maximum foraging distance (km). In both cases, we included food abundance (seeds·m^-2^), percentage of flooded rice paddies (%), percentage of flooded rice paddies with standing stubble (%), percentage of ploughed and flooded rice paddies (%), rain (l·m^-2^), minimum temperature (°C), wind speed (km·h^-1^), moonlight brightness (%), and Julian date (days after 1 November) as potential predictive variables. To test for potential collinearity among predictors, we calculated Pearson's correlation coefficients, and removed highly correlated covariates (*r* > 0.50). Prior to analysis, we log-transformed rain, wind speed and minimum temperature, and arcsine-transformed flooded area and moonlight. Given the high correlation among covariates, we only kept in the analysis percentage of flooded rice paddies, minimum temperature, wind speed and moonlight brightness. We also included bird ID as a random effect, since locations from the same individual are not independent.

To perform the RSF analysis we used a GLMM with a binomial distribution and a logit-link function. Our binary response was used (1) versus non-used (0) rice paddies. Straw manipulation (two levels: standing stubble and straw incorporated into the soil) and pebble size (three levels: < 0.5 cm, 0.5–1.0 cm, and > 1.0 cm) were included as fixed factors. Food abundance (seeds·m^-2^), water depth (cm), paddy size (ha), Julian date, distance to diurnal roosting site (km), and distances to the closest power line (km), paved road (km), country road (km) and urban area (km), were included as covariates. Distance to the closest urban area, country road, and Julian date were excluded from the model due to collinearity issues (see above procedure). Candidate models were built using all possible combinations of explanatory variables. Models were evaluated using the Akaike information criterion with a correction for small sample sizes (AICc; [65]). Models within 2 units of ΔAICc were considered equally good. In the RSF analysis, we also performed ‘full-model averaging’ of the subset of models with ≤ 0.95 accumulated weight [65], an appropriate approach when there is uncertainty in the selection process (i.e. the best model has a low weight; [66]). We further calculated the relative importance (RI) of each variable as the sum of the weight of the models where that variable was present. For all the models we used the lme4 package in R3.3.3 [67]. All values are expressed as a mean ± standard error.

## Results

### Space use

Northern pintails followed a common routine throughout the winter: they left the diurnal roosting site 16.1 ± 1.9 min after sunset, spent the night in the foraging areas (flooded rice fields), and left them 29.7 ± 1.2 min before dawn to go back to the diurnal roosting site. Overall, birds showed high site fidelity to the foraging area and came back to the same rice paddy during four or five consecutive days.

**Fig 3:**
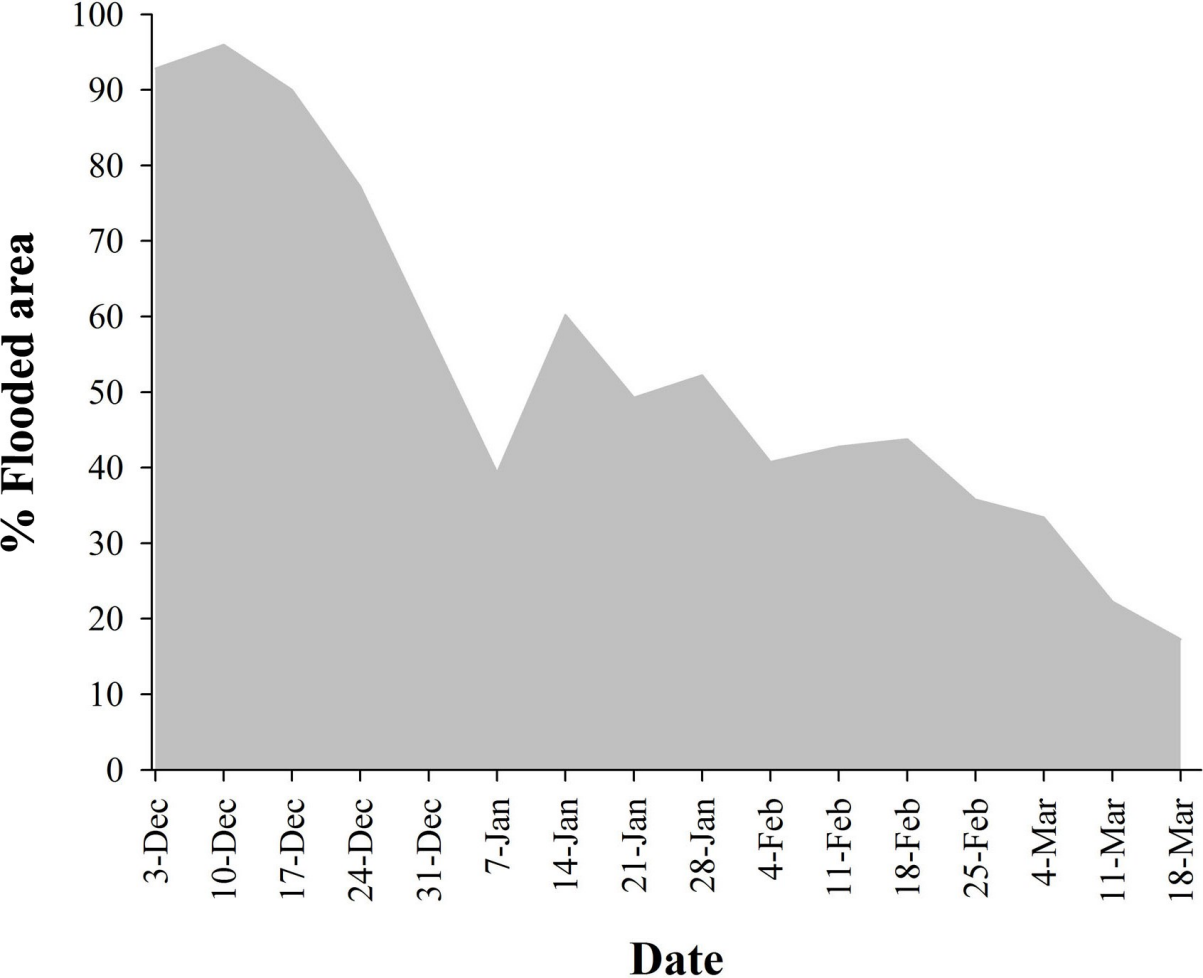
Variation in the proportion of flooded area (rice paddies with and without standing stubble) through the winter season in a 20 km radius from the diurnal roosting area. The total flooded area in late winter was a 75.1% lower than in early winter.

**Fig 4:**
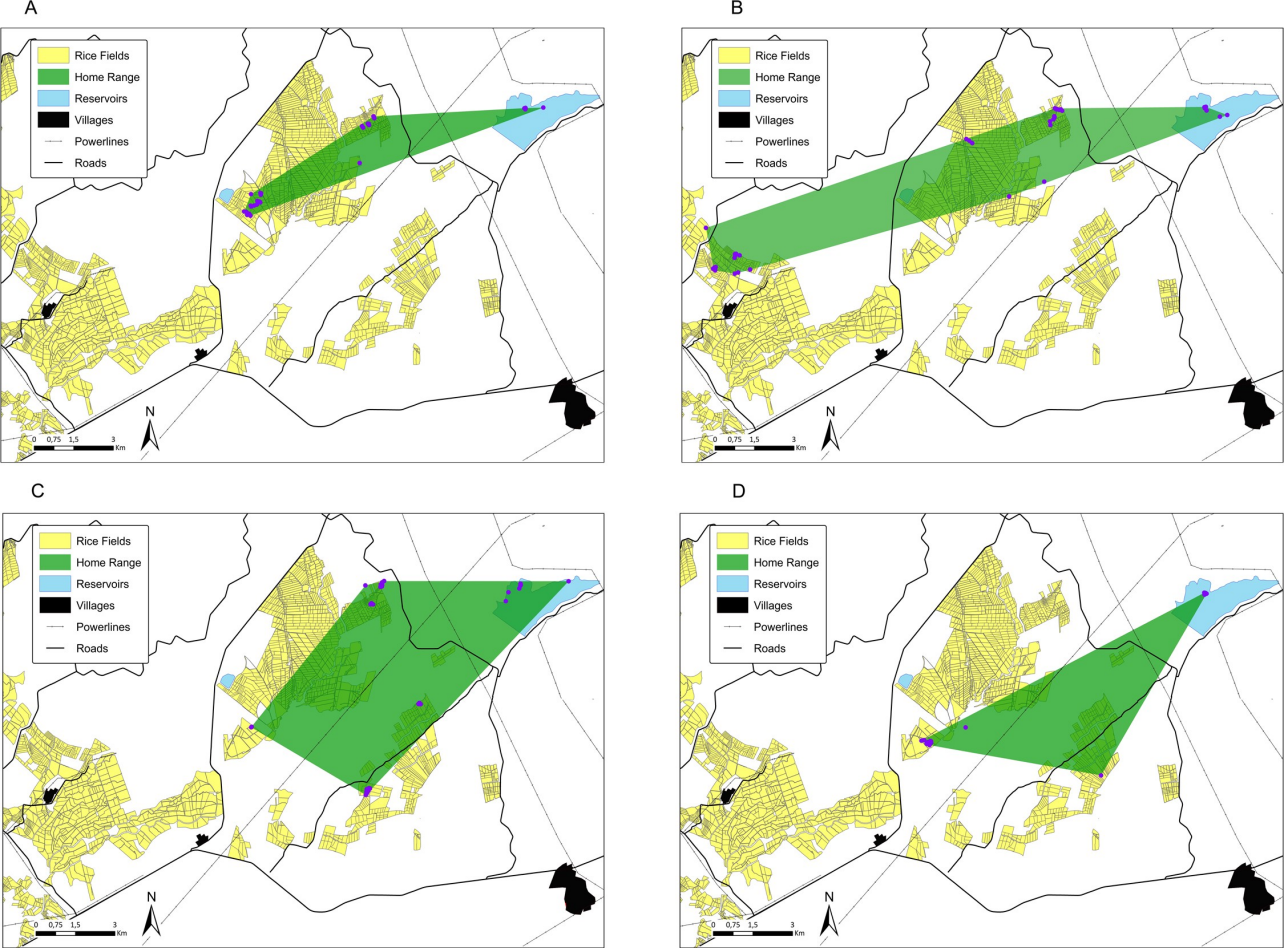
Examples of nocturnal home-ranges of northern pintails overwintering at Extremadura rice fields. Panels A and B: the Pint 06’s MCPs during the second week of December 2012 (1,312 ha; 96% of rice paddies were flooded and moonlight brightness 6%) and during the second week of January 2013 (4,082 ha; 39% of rice paddies were flooded and moonlight brightness 10%). Panels C and D: the Pint 05’s MCPs during the fourth week of January 2013 (4,765 ha; 45% of rice fields flooded and moonlight brightness 88%), and during the second week of January 2013 (2,637 ha; 39% of rice fields flooded and moonlight brightness 10%).

MCP and maximum foraging distance were 9,652.6 ± 1,588.9 ha (n = 78) and 11.4 ± 0.6 km (n = 78), respectively. In the MCP analysis, the best-supported model included the proportion of flooded rice fields and brightness of moonlight (Table 1 and Fig 3). The MCP model indicated that northern pintails’ home-range was larger when the proportion of flooded rice fields was lower (Fig 4A and 4B) and the moonlight was more intense (Table 1 and Fig 4C and 4D). The best model for maximum foraging distance showed that the proportion of flooded rice fields had a positive effect on the travelled distance (w_i_ = 0.457; Table 1). Thus, the less proportion of flooded paddies, the further the dabbling ducks went. The top-ranked candidate models explaining space use by northern pintails through the winter did not include minimum temperature (4.29 ± 0.64°C in 2012–2013; 4.24 ± 0.72°C in 2014–2015).

**Table 1:** Summary of the GLM models explaining spatial use by northern pintails during night-time.

Model variables	df	logLik	AIC_C_	ΔAIC_C_	w_i_
**Home-range (MCP)**					
Flooded rice paddies + Moonlight	5	-121.135	253.1	0.00	0.358
Flooded rice paddies + Moonlight + Wind speed	6	-120.481	254.1	1.04	0.213
Flooded rice paddies	4	-123.011	254.6	1.47	0.172
**Maximum foraging distance**					
Flooded rice paddies	4	-38.814	86.2	0.00	0.457
Flooded rice paddies + Moonlight	5	-38.211	87.3	1.08	0.266

Nocturnal home-range (defined using minimum convex polygon; MCP) and maximum foraging distance models are shown separately. Models were sorted using the Akaike information criterion with a correction for small sample sizes (AICc), the increase in AICc compared to the best model (ΔAIC_C_), the weight of each model (w_i_), and the values of the -2log-likelihood (logLik) function. Only models following ΔAIC_C_ < 2 are displayed.

### Resource selection

The four top-ranked candidate models explaining rice paddy selection by foraging northern pintails included water depth, standing stubble and pebble size (Table 2). Of these, three also included food abundance, and one included distance to paved roads and rice paddy size (Table 2). There was high model selection uncertainty (the best AIC model was not strongly weighted; w_i_ = 0.136); thus, inference was based on all models in the candidate set using full-model averaging. Estimates from the model averaging showed that water level (14.10 ± 0.31 cm depth) and stubble presence had a strong influence on rice paddy selection (Table 3). Moreover, presence of pebbles larger than 0.5 cm in diameter had a negative effect on rice field selection by northern pintails (Table 3). Food abundance was present in three of the four best-ranked models (Table 2); nonetheless, its effect was only marginally significant when full-model averaging was performed (Table 3).

**Table 2:** Summary of the GLM models explaining resource selection by northern pintails during night-time.

Model variables	df	logLik	AIC_C_	ΔAIC_C_	w_i_
Pebble size + Food abundance + Water depth + Straw	7	- 102.337	219.1	0.00	0.136
Pebble size + Distance to road + Food abundance + Water depth + Straw	8	- 101.595	219.8	0.65	0.098
Pebble size + Food abundance + Paddy size + Water depth + Straw	8	- 101.791	220.2	1.04	0.081
Pebble size + Water depth + Straw	6	- 104.341	221.0	1.89	0.053

Models were sorted using the Akaike information criterion with a correction for small sample sizes (AICc), the increase in AICc compared to the best model (ΔAIC_C_), the weight of each model (w_i_), and the values of the -2log-likelihood (logLik) function. Only models following ΔAIC_C_ < 2 are displayed.

**Table 3:** Estimates for the averaged resource selection model (RSF, using models within an Akaike accumulated weight ≤ 0.95) and relative importance of the parameters (RI).

Variable	β	SE	RI	*P*
Water depth	- 0.96	0.27	1.00	< 0.001
Straw	4.64	1.46	1.00	0.001
Pebble size	- 1.56	0.62	0.97	0.011
Food abundance	0.49	0.25	0.75	0.053
Distance to road	- 0.38	0.34	0.40	0.254
Paddy size	0.30	0.34	0.33	0.376
Distance to power line	- 0.21	0.44	0.28	0.640
Distance to roosting site	- 0.06	0.32	0.24	0.857

Variables are sorted by the *P*-value.

## Discussion

Winter management of rice fields can have important consequences for the conservation of migratory waterbirds worldwide [16]. Our GPS-GSM data revealed that northern pintails roosted during daylight in a reservoir near the foraging areas visited at night. Home-range area and maximum foraging distance from this reservoir were mainly influenced by the surface of flooded rice fields and moon phase. Northern pintails selected to forage flooded rice paddies were stubble was present and were pebble size was relatively small. By contrast food abundance and other landscape variables were not strong predictors of rice paddy use at night. These results are essential to delineate appropriate management strategies in rice fields that underpin conservation efforts of migratory dabbling ducks [68]. Nevertheless, owing to the relatively small-sample size, our findings should be interpreted with caution.

Northern pintails’ home-ranges were up to 35,000 ha. These home-ranges are larger than those of northern pintails using coastal marshes in West Europe [69] as well as of other ecologically similar dabbling ducks [70]. Home-range size and maximum foraging distance increased with decreasing surface of flooded rice paddies. Soil softening, seed hydration and water availability are key to efficient feeding by dabbling ducks relying on rice seeds [59,71,72]; thus, as the surface of flooded rice paddies decreased, northern pintails were probably forced to increase their home-ranges to find adequate foraging grounds. We also showed a consistent (positive) relationship between moonlight and the spatial use of rice fields by northern pintails. Previous studies have shown that waterbirds can take advantage of the moonlight to increase their exploratory activity to find new foraging areas [73,74].

Water depth and stubble presence were the most important predictors of rice paddy selection by northern pintails. Previous studies performed in rice fields showed that depths of 14–22 cm lead to the greatest densities of dabbling ducks during daylight [75], and overall, suitable water depth for dabbling ducks in rice fields is assumed to be > 16 cm [76]. These water levels fall within the water depth range (9–21 cm) of flooded rice paddies used by northern pintails at Extremadura rice fields. On the other hand, studies based on nocturnal counts in rice fields in Japan [29] and France [30] also found that rice paddies with stubble harboured higher abundances of dabbling ducks. This positive association seems to be caused by the higher density of rice seeds available where stubble was present, compared to ploughed paddies (see [30] and references therein). Moreover, it has been suggested that foraging in paddies with standing stubble could enhance crypsis [77,78], which could compensate the reduction in the ability to detect predators during nocturnal foraging [79].

By contrast we found no evidence that northern pintails habitat selection patterns during nocturnal foraging were affected by the proximity to urban areas, paved roads or power lines. These results are consistent with other studies that did not detect an effect of these landscape variables on the use of foraging areas by migratory waterbirds [48,80,81]. Nevertheless, power lines can influence the choice of nocturnal roosting sites in some waterbird species, such as the black-tailed godwit *Limosa limosa* [46]. It is important to note that, in our study area, population density is relatively low (9 villages, with 200–1800 people each; Fig 1), infrastructures are scarce, and northern pintail hunting is forbidden (hunting of other duck species is unusual too).

Noticeably, ducks avoided paddies where pebbles > 0.5 cm were abundant [56]. Dabbling ducks generate a water flow through a lamellar structure in the margins of the bill [57], a feeding mechanism that could be impaired by large pebbles and thus reducing foraging efficiency [56]. The greater presence of these pebbles in the superficial soil layer is related to soil properties but also to pre- and post-harvest treatments [82]. During these treatments, the soil is compacted to form an impermeable layer (i.e. hardpan). This layer avoids root penetration and water loss by percolation and increases rice capacity to efficiently use water and nutrients [83]. On top of the hardpan, farmers create a fertile layer with a high content of organic matter and nutrients, where the roots of the rice develop [84]. When this fertile layer is too thin, ploughing can increase the number of big pebbles in the upper part of this layer [85,86], thus transforming a potential foraging area into an unavailable one, at least for dabbling ducks. Our results can be applied to improve habitat features for dabbling ducks in the world's most important crop [11,87]. As in other important rice fields for overwintering waterbirds, rice seed density is not a limiting resource in our study area [30,31], thus management of rice paddies arise essential to integrate a worldwide productive crop into conservation efforts of migratory dabbling ducks.

### Management implications

Plans to benefit non-breeding migratory waterfowl in Western Europe have focused on conserving diurnal roosting areas, but nocturnal foraging areas are mostly unprotected and unmanaged [25,31]. In Extremadura, some reservoirs have been designated Special Protection Areas owing to high numbers of dabbling ducks they support during daylight, but adjacent rice fields where ducks forage at night are unprotected [31]. Our results reveal benefits of foraging areas used at night that should be conserved in the rice agroecosystem. Large surfaces of rice fields are currently being converted to other crops. In Extremadura 6,000 ha have been replaced by fruit, almond, and olive tree crops [88]. Reductions in personal income tax by the Spanish government for rice farmers may lessen the land conversion to other crops (BOE-A-2017-13896/Orden HFP/1159/2017). Further, plans for waterbird conservation could be included as part of the greening measures of the Common Agriculture Policy and as a condition for a reduced tax rate thereby increasing a farmer’s commitment to sustainable farming.

We recommend the following actions to benefit migratory waterfowl. (1) Farmers should maintain flooded and unploughed rice paddies throughout winter, which will not incur any costs [10,72,89]. In Extremadura and similar areas this would entail keeping paddy drains closed throughout the winter to retain as much water as possible; (2) Establish an optimal water depth (i.e., 9–21 cm) for management in flooded fields after harvest [90,91]; (3) In paddies with pebbles > 0.5 cm diameter, create a fertile layer 40–50 cm thick and level soil every 3–4 years to reduce pebbles at the soil surface as a result of previous ploughing [84,85]; and (4) Because other waterbird species (e.g., geese, shorebirds or cranes) forage in rice fields and have different modes of feeding from dabbling ducks (e.g. [91–93]), their needs should be integrated into overall management plans.

Lastly, we point out gaps in knowledge and suggest to research the nocturnal behaviour of a variety of species would go a long way to developing our understanding of not only interactions between waterbirds and rice cultivation but of how best to find a middle ground with conservation and land use practices [26,27,60].

## Supporting information

S1 Data Space use data file. (XLSX)

S2 Data Resource selection data file. (XLSX)
